# A Dual Route Model for Regulating Emotions: Comparing Models, Techniques and Biological Mechanisms

**DOI:** 10.3389/fpsyg.2020.00930

**Published:** 2020-06-04

**Authors:** Alessandro Grecucci, Irene Messina, Letizia Amodeo, Gaia Lapomarda, Cristiano Crescentini, Harold Dadomo, Marta Panzeri, Anthony Theuninck, Jon Frederickson

**Affiliations:** ^1^Clinical and Affective Neuroscience Lab, Department of Psychology and Cognitive Sciences, University of Trento, Rovereto, Italy; ^2^Department of Psychiatry and Psychotherapy III, Ulm University, Ulm, Germany; ^3^Department of Languages and Literatures, Communication, Education and Society, University of Udine, Udine, Italy; ^4^Department of Neuroscience, University of Parma, Parma, Italy; ^5^Parma Schema Therapy Center, Parma, Italy; ^6^Department of Developmental Psychology and Socialisation, University of Padua, Padua, Italy; ^7^Oxleas NHS Foundation Trust, London, United Kingdom; ^8^Washington School of Psychiatry, Washington, DC, WA, United States

**Keywords:** emotion regulation, cognitive-behavioral therapy, schema therapy, experiential-dynamic therapy, memory reconsolidation

## Abstract

The aim of this article is to present recent applications of emotion regulation theory and methods to the field of psychotherapy. The term Emotion Regulation refers to the neurocognitive mechanisms by which we regulate the onset, strength, and the eventual expression of our emotions. Deficits in the regulation of emotions have been linked to most, if not all, psychiatric disorders, with patients presenting either dysregulated emotions, or dysfunctional regulatory strategies. We discuss the implications of regulating emotions from two different theoretical perspectives: the Cognitive Emotion Regulation (CER), and the Experiential-Dynamic Emotion Regulation (EDER) model. Each proposes different views on how emotions are generated, dysregulated and regulated. These perspectives directly influence the way clinicians treat such problems. The CER model views emotional dysregulation as due to a deficit in regulation mechanisms that prioritizes modifying or developing cognitive skills, whilst the EDER model posits emotional dysregulation as due to the presence of dysregulatory mechanisms that prioritizes restoring natural regulatory processes. Examples of relevant techniques for each model are presented including a range of cognitive-behavioral, and experiential (including both dynamic and cognitive) techniques. The aim of the paper is to provide a toolbox from which clinician may gain different techniques to enhance and maintain their patient’s capacity for emotional regulation. Finally, the biological mechanisms behind the two models of emotion regulation are discussed as well as a proposal of a dual route model of emotion regulation.

## Introduction

When interacting with others, emotions are constantly generated and guide our interpersonal experiences. Sometimes, regulating our emotions and emotionally driven behaviors is essential for creating healthy relationships. Emotion regulation refers to the capacity to modulate some aspects of our emotional experiences (Gross, 1998; Grecucci et al., 2013a,b,c; Grecucci et al., 2017). Although emotion regulation was initially intended as a field of basic research, clinicians quickly understood its relevance for their practice. Studying the mechanisms involved in the regulation of emotions is particularly relevant when considering the failure to regulate interpersonal emotions and emotionally driven behaviors that characterize psychiatric disorders (Kring and Werner, 2004; Ochsner and Gross, 2008; Grecucci, 2012; Giorgetta et al., 2012, 2014; Grecucci et al., 2015b, 2019; Messina et al., 2016; De Panfilis et al., 2019; Sorella et al., 2019). Nearly all psychiatric disorders include one or more primary dysregulated emotions (e.g., anger in borderline personality disorder, sadness in depression, fear in anxiety disorders, shame in narcissistic personality disorders, etc.), or detrimental regulatory strategies (avoidance of feared situations in anxiety disorders, rumination in depression or excessive control of bodily signals in hypochondria). Thus, the ability to regulate emotions adaptively is essential for well-being. The ubiquitous presence of emotion dysregulation in psychopathology may suggest a potential unique focus for intervention across disorders. Consistently, the importance of emotion regulation is now recognized by numerous psychotherapy approaches. Despite this general agreement, different routes can be observed in the conceptualization of emotion regulation and in the associated therapeutic techniques used in clinical practice.

### Cognitive Emotion Regulation Model (CER)

Cognitive-behavioral psychotherapists train their patients in using attentional, cognitive and behavioral strategies to promote their ability to cognitively control their emotions. This view is consistent with the most popular model of emotion regulation proposed by Gross (1998, 2014), and extensively investigated in affective neuroscience (Ochsner et al., 2002; Ochsner and Gross, 2005; Grecucci et al., 2013a,b,c). Gross’ “Process Model of Emotion Regulation” is based on appraisal theory (Frijda, 1986; Scherer et al., 2001) and relies on the assumption that events lead to cognitive appraisals that generate emotional responses. According to CER, emotions are generated through the following sequence: (1) an individual is exposed to a situation (Situation); (2) he/she attends to a particular aspect of the situation (Attention); (3) interprets the situation (Appraisal); and (4) produces an emotional response at a behavioral, subjective and physiological level (Response). Gross suggests that emotion regulation or dysregulation can occur within any of the five stages or points of the process model through use of the following strategies at each point: (1a) situation selection, (1b) situation modification, (2) attentional deployment, (3) cognitive change, (4) response modulation. In this view, the main mechanism of dysregulation is the lack of, or failure to apply, an appropriate regulatory strategy and every emotion can in principle become dysregulated. We make the point, that the field of cognitive-behavioral therapies from Beck (2011) to third wave therapies like Dialectical Behavior Therapy Linehan, 1993a, b) use interventions for emotional regulation that fit with the CER model.

### Experiential-Dynamic Emotion Regulation Model (EDER)

Differently from CBT, other approaches such as psychodynamic therapy (Gabbard, 2014) and humanistic therapies (Perls et al., 1951; Rogers, 1951; Berne, 1961), and new approaches such as schema therapy (Young et al., 2003) and emotion-focused therapy (Greenberg, 2004), largely employ experiential techniques that work on emotion themselves by recreating real experiences that allow the expression of feelings, attitudes, and beliefs, instead of their control. As a matter of fact, individuals can actively regulate their emotional state by allowing themselves to experience it, focusing on their bodily sensations and becoming aware of the emotional state itself (Vandekerckhove et al., 2011, 2012). Within this framework, experiencing emotions is critical in that it involves overcoming avoidance, leading individuals to learn that they are more able to cope with their most painful feelings and emotions after having faced them and survived to them (Greenberg and Bolger, 2001). Some theorists also suggest that experiential or bodily felt feeling, together with affective self-awareness, may be considered as more beneficial than an overall cognitive focus (Vandekerckhove et al., 2012; Vandekerckhove and Wang, 2018). Experiential techniques cannot be explained by the CER model. Thus, a different framework has been proposed called “Experiential-Dynamic Emotion Regulation” model (EDER, Grecucci, 2012; Grecucci et al., 2015b, 2016; Frederickson et al., 2018). In the EDER model, emotions are posited to be prewired and partially independent of cognition: situations trigger emotional responses (subjective and physiological reactions) from which a coherent expression-action is produced. These approaches are grounded in affective neuroscience findings (Panksepp, 1998; Damasio, 1999; Panksepp and Biven, 2012; Herwig et al., 2010; Lutz et al., 2012), which consider that emotions generated by mainly subcortical areas (Panksepp and Biven, 2012), rise, peak, and then go flat with a Gaussian-like shape, all this proportional to the nature and intensity of the stimulus (Grecucci and Job, 2015; see also Campos et al., 2004 for a similar account). According to this view, no voluntary effort is required to regulate emotions and emotions are not inherently dysregulated (Frederickson et al., 2018). Once experienced, an emotion informs cognitive appraisal of the situation and can be expressed and channeled into healthy actions (Frederickson, 2013. In line with psychodynamic and affective neuroscience principles, we point out the idea that when dysregulation is present, it is due to: (1) excessive conditioned anxiety (anxiety triggered by the perception and/or experience of an emotion); or (2) defense mechanisms which create defensive affects (Frederickson, 2013) (or secondary emotions). EDER defines both cases of “excessive anxiety” and “defensive affects” as Dysregulated Affective States (DAS). In this framework emotional regulation is achieved by removing dysregulatory mechanisms (conditioned anxiety and defensive affects, DAS), as opposed to adding or learning new regulatory strategies (as prescribed by CER, see Table 1). Once dysregulatory mechanisms are removed, the patient is able to experience the underlying primary emotion (Coughlin della Selva, 1996; McCullough, 1997; Frederickson, 2013). Moreover, while CER assumes that every emotion is subjected to abnormal up- or down-regulation, EDER holds that primary emotions (or emotions reactive to the event) are to be facilitated in their experience/expression (even up-regulated where needed), and secondary emotions (anxiety and defensive emotions, i.e., DAS) are to be down-regulated (or even blocked). Differences between CER and EDER models are summarized in Table 1. In the next sections, we present in detail these two frameworks providing examples of different emotion regulation technique proposed in the context of different psychotherapies. With this effort, our aim is to provide a toolbox from which clinician may gain different techniques to enhance and maintain their patient’s capacity for emotional regulation, and stress their relation with emotion regulation problems. Indeed, some of the techniques presented are taken from the previous literature, but their relation with emotion regulation and dysregulation has not fully explored yet.

**TABLE 1:** Comparing two models of emotion regulation.

	Cognitive emotion regulation model	Experiential-dynamic emotion regulation model
Emotion generation	Conscious cognitive appraisal produces emotions	Emotion is automatically generated by subcortical structures with certain properties
Emotion regulation in normality	Voluntarily cognitive top-down regulatory strategies at different levels (situation selection, situation modification, etc.)	The brain self-regulates emotions through a biological mechanism that return them to baseline
Emotion dysregulation	Failure to use regulatory strategies	After emotion is generated, dysregulatory mechanisms intervene that stop self- regulatory
What is dysregulated	Every emotion in principle is down- or up-regulated	Anxiety and defensive affects are to be down-regulated (or blocked)
Therapeutic strategy	The clinician teaches the patient emotion regulation strategies	The clinician helps the patient to remove dysregulatory mechanisms and to down- regulate DAS
Treatment modalities	Cognitive-Behavioral Therapies, Dialectical Behavior therapy, Acceptance and Commitment Therapy	Experiential-Dynamic and Experiential-Cognitive Therapies
Biological mechanism behind regulation	Extinction	Memory Reconsolidation
Processing	Top-down, voluntary, explicit, conscious, verbally mediated	Bottom-up, not voluntary, implicit, unconscious, independent from language
Neural basis	Mainly cortical and lateralized at left	Mainly subcortical and lateralized at right

With this effort, our aim is to provide a comprehensive theoretical framework based on emotion regulation theories for the conceptualization of therapeutic techniques coming from different psychotherapy approaches. A sort of toolbox from which clinician may gain different techniques to enhance and maintain their patient’s capacity for emotional regulation.

## Cognitive-Behavioral Techniques to Regulate Emotions

In this section we focus on therapeutic techniques developed as part of CBT approaches which are consistent with CER model. We make the point that these techniques are based on the view of emotion dysregulation as a lack of regulatory strategies coherently with CER model. In line with the Process Model part of the CER model (Gross, 1998), emotion dysregulation is treated through teaching the patient with: (i) behavioral strategies (e.g., selecting and exposing to appropriate situations and adaptive situation modification, point 1a and 1b in the Process Model); (ii) attentional strategies (e.g., distraction from negative cues, concentration on positive cues, point 2); (iii) cognitive methods (e.g., cognitive restructuring, point 3); and (iv) response modulation (e.g., applied relaxation). In CBT approaches, several techniques have been described, correspondent to the different phases of the Process Model proposed by Gross (1998).

### Cognitive Change (or Reappraisal)

Cognitive change is the core of Cognitive Behavior Therapy (CBT) approaches. According to this approach, emotion follows cognition and are directly influenced by them. In other words, as Greenberger and Padesky (2015) phrase it “changing cognition changes emotions” and “It’s the thought that counts.” Thus, many CBT strategies serve the aim of changing the appraisal of a stimulus so that a different emotional or mood outcome can be achieved. A distressing emotional state can thereby be regulated when the person is able to adjust their perspective on the stimulus that distresses them (cognitive top down modulation over emotions). To this aim the intervention require the following steps:

1. The starting point in CBT is measuring symptoms and distress across different situations (Hawton et al., 1989; Westbrook et al., 2011). The therapist helps the patient to focus on the stimulus that precedes the emotion to find the maladaptive appraisal that generated that dysregulated emotion. The patient may be asked to focus on what thoughts are distressing or symptomatic for them.
2. The next stage within the CBT protocol involves more detailed *identification of the cognitions* or thoughts associated with the problematic symptoms. Thoughts about feelings, thoughts about thoughts, thoughts about physiology or behavior are identified and monitored across different situations (Beck, 2011; Westbrook et al., 2011). A variety of techniques are used that may include keeping a thought diary whilst exposed to different situations or using role play or imagery during a therapy session to improve thought recollection associated with the problematic situations. A technique called “downward chaining” (Beck, 2011), where a patient is repeatedly asked “What is so bad about that?”, is used to uncover deeply held beliefs about self, other and the world that may be producing symptoms.
3. The previous steps are aimed to reach the core of CBT intervention: *changing appraisals or thoughts* (or Reappraisal). Several techniques may be used to promote reappraisal:
  (a) *Socratic questioning* (Beck and Emery, 1985; Westbrook et al., 2011), also termed *guided discovery* (Kuyken and Padesky, 2011). This is a questioning style that seeks to uncover how the patient thinks and arrives at their distressing conclusions, and supports the patient to take different perspectives to reach less distressing conclusions. Greenberger and Padesky (2015) have added socratic questions into their thought diaries to prompt patients to question their automatic thinking styles. Lists of ready-made “cognitive distortions” or “thinking errors” are often used as short hand to help patients identify symptom-maintaining thoughts.
  (b) *Role playing or guided imagery* to help the patient rescript a particular scenario in order to arrive at a new appraisal. Coping statements may be prepared by therapist and patient that serve as ready-made alternative appraisals which the patient can remind themselves of during problematic situations.
  (c) *Behavioral experiments* within CBT support cognitive reappraisal by investigating evidence to support one thought as opposed to another thought (different from behavioral experiments in behavioral approaches that aims to habituation to a problematic stimulus and extinguish a conditioned response). Such experiments could be active (trying new behaviors) or observational (seeing what happens in different situations).

### Response Modulation

Physical techniques (Westbrook et al., 2011) are focused on regulating levels of physiological arousal and include various forms of relaxation, controlled breathing and physical exercise. These techniques either raise the patient’s arousal level so that they are alert enough to engage their thinking, or lower the arousal level that impedes clear thought. Physical techniques primarily address anxiety symptoms. However, these techniques are used sparingly since physical techniques may maintain dysfunction when they are used to avoid thought change. For example, a patient might use breathing techniques to reduce distress without examining and changing their distress-maintaining thoughts.

### Mindfulness-Based Techniques

We include mindfulness among CBT techniques, although it is now used by many psychotherapy approaches, compared to the previous techniques. Mindfulness principles are increasingly being applied in the so-called third generation CBTs, such as the Acceptance and Commitment Therapy (ACT) (Hayes et al., 2011), Dialectical Behavior Therapy (DBT) (Linehan, 1993a, b), Mindfulness-based cognitive therapy (MBCT) (Segal et al., 2012), Compassion-focused Therapy CFT (Gilbert, 2009). Mindfulness-based methods teach patients to either focus their attention on specific meditation objects, typically breath or bodily sensations, with a non-judgmental attitude and receptive awareness of any thought, emotion or sensation that occurs moment-to-moment (Linehan, 1993a; Hayes et al., 2011; Lutz et al., 2012; Segal et al., 2012). Thus, it involves several aspects of emotion regulation: attentional deployment (for example to bodily sensations instead that to stressful stimuli), cognitive change (non-judgmental approach to emotions) and response modulation (non-reaction attitude). An important aspect of mindfulness-based practice is that patients learn how to detach and be less absorbed in unhelpful thoughts and emotions; in particular to experience thoughts as products of the mind, i.e., thoughts are what they are (thoughts), not what they say they are (facts). This process is referred to as cognitive defusion or reperception (Hayes et al., 2011) or also intimate detachment or decentering (see in Grecucci et al., 2015a) (Table 2).

**TABLE 2:** Standard Cognitive-Behavioral techniques for regulating emotions.

Mode	Regulatory process (according to CER)	Strategy/Intervention	Model of therapy
Behavioral	Response modulation	Progressive muscle relaxation, diaphragmatic breathing	CBT
Behavioral	Situation selection, appraisal	Behavioral activation, systematic desensitization	CBT
Behavioral	Situation selection, attention, appraisal, response modulation	Exposure and response prevention, behavioral experiments	CBT
Behavioral	Situation modification, appraisal, response modulation	Opposite action	DBT, ACT
Cognitive	Appraisal	Psychoeducation and normalization	All CBT-derived therapies
Cognitive	Attention, Appraisal, Response modification	Monitoring thoughts and feelings through diary keeping, Problem solving, weighing pros and cons	CBT, DBT, ACT
Cognitive	Appraisal	Image rescripting	CBT
Mindfulness-based	Attention, response modulation	Distraction, mindfulness meditation, thought defusion and attitudes, Compassionate imagery	MBSR, MBCT, ACT, DBT, MBRP

*CER, cognitive emotion regulation; CBT, cognitive-behavioral therapy; DBT, dialectical behavioral therapy; ACT, acceptance and commitment therapy; MBSR, mindfulness-based stress reduction; MBCT, mindfulness-based cognitive therapy; MBRP, mindfulness-based relapse prevention.*

## Experiential-Dynamic Techniques to Regulate Emotions

Beyond the context of CBT traditional approaches, the CER model of emotion regulation has been considered as just one way for the conceptualization of therapeutic intervention on emotion regulation (see for example Messina et al., 2016). For example, cognitive and attention resources required for the CBT techniques described above may be depleted and unavailable for optimal use when strong emotional activation occurs (Opialla et al., 2015). Indeed, studies on how individuals choose regulatory strategies observed that reappraisal strategies are more likely to be chosen for low intensity emotional stimuli (Sheppes et al., 2011; Sheppes, 2014). Moreover, in some cases, the cognitive control of emotion can have negative consequences. An example are the well-described paradoxical effects of cognitive control on the incursion of unwanted thoughts (Wegner et al., 1987; Abramowitz et al., 2001). Additionally, some theorists consider cognitive control-based approaches as a “narrow view” in emotion regulation research (Greenberg and Vandekerckhove, 2008), since they generally imply an emotion generation system and a subsequent one, aimed at regulating emotions. According to this perspective, when an emotion arises, it consequently has to be regulated. Conversely, experiential therapies suggest a “broader view,” in which emotions can be inherently regulated as well as regulatory. Experiential therapies – such as the emotion-focused one (Greenberg, 2004) – not only involve accessing the primary emotions often underlying secondary symptomatic ones, but also the awareness, experience and tolerance of underlying emotions as a first step in the subsequent reduction of negative affect in the longer run (Hunt, 1998).

In the present section, we describe experiential techniques aimed to regulate emotions, which extend the options of intervention for the therapist. Experiential techniques are strategies designed to promote the transformation of emotions, as opposed to strategies that target processes that are related to emotions (thinking, attention, behavior). Experiential techniques have been mainly proposed as part of modern approaches of psychodynamic psychotherapy such as Intensive Short-Term Dynamic Psychotherapy, ISTDP (Davanloo, 1990, 2000; ten Have-de Labije and Neborsky, 2012; Frederickson, 2013; Abbass, 2015; Messina et al., 2020), Accelerated Empathic Dynamic Psychotherapy, AEDP (Fosha, 2000), Affect Phobia Therapy, APT (McCullough, 1997); and Intensive Experiential Dynamic Psychotherapy, IE-DP, Osimo, 2003). We refer to ER techniques coming from these approaches as *experiential-dynamic techniques*. Other experiential techniques have been proposed as part of third wave cognitive approaches, such as Schema Therapy (Young et al., 2003) and Acceptance and Commitment Therapy (Hayes et al., 2011). We describe this second category of experiential techniques within the category *experiential-cognitive techniques*. Despite their differences, both categories involve psychological processes which are not contemplated in the CER model, whereas they may find a common theoretical basis in the EDER model of emotion regulation.

In the field of experiential dynamic therapies and in accordance with recent findings in Affective Neuroscience (Panksepp and Biven, 2012), emotions are understood to be generated through subcortical neuroperception of the environment and the experience of stimuli in reality (Davanloo, 1990, 2000; Benjamin, 1993; Coughlin della Selva, 1996; Panksepp, 1998; Damasio, 1999; Frederickson, 2013; Pappaianni et al., 2019). Emotions are hard-wired at birth (Panksepp, 1998) with inborn adaptive action tendencies (Frijda, 1986). Primary feelings (directly elicited by the stimulus, and not by defenses) motivate us to take effective action in response to a situation (Damasio, 1999; Grecucci et al., 2015b). They allow us to assert ourselves when wronged, to celebrate when victorious, and to grieve when we experience loss. So why do seemingly adaptive emotions become dysregulated? Following Experiential-Dynamic Therapy (EDT) principles we propose two primary causes: (1) true emotion can be paired with excessive anxiety due to conditioning (Davanloo, 1990, 2000; Coughlin della Selva, 1996; Frederickson et al., 2018); or (2) the patient uses defenses which create dysregulated emotions (Davanloo, 1990, 2000; Coughlin della Selva, 1996; Frederickson et al., 2018). We define both as Dysregulated Affective States, DAS (Frederickson et al., 2018). From an experiential-dynamic point of view, the therapist must help the patient to both regulate anxiety and let go of the defenses that create (secondary) defensive affects causing the DAS-associated symptoms. Then the patient must face the underlying feelings that triggered anxiety, that may be even up-regulated. From evolutionary theory (Panksepp, 1998) we understand that life situations trigger feelings that mobilize adaptive action (Frijda, 1986). However, coherently with an attachment perspective (Bowlby, 1969, 1973, 1980), children experiencing these emotions may discover that experiencing and expressing their feelings threatens the security of their relationships, which is signaled to the child by a rise in anxiety (signaling an internal and interpersonal danger). The combination of emotion and overwhelming anxiety creates a dysregulated affective state (DAS) due to excessive conditioned anxiety (Grecucci et al., 2015b; Frederickson et al., 2018).

### Anxiety Regulation

To assess and regulate anxiety, dynamic approaches pay attention to the bodily symptoms of anxiety which are correlated with patterns of activation of the somatic and autonomic nervous systems. Low level anxiety is discharged into the somatic nervous system (the striated muscles, Davanloo, 2000): the patient becomes tense, gets tension headaches, and sighs when the therapist explores feelings. When anxiety rises too high, the somatic nervous system turns off, and the parasympathetic nervous system turns on, yielding symptoms of nausea in the stomach, sudden needs for defecation and urination, stomach aches, and migraine headaches. Now anxiety must be regulated. When anxiety rises even higher, the activation of the parasympathetic nervous system becomes even more extreme, causing cognitive/perceptual disruption (Davanloo, 2000). Now the patient suffers dizziness, blurry vision, ringing in the ears, and problems in thinking and perception. Now anxiety regulation is imperative. The experiential dynamic therapist explores emotions to help the patient develop the capacity to bear her feelings at progressively higher levels without anxiety (Frederickson, 2013; Abbass, 2015). The therapist explores feelings until anxiety is too high. Then the therapist regulates anxiety. Then feelings are gradually explored again. In this stepwise exposure method, the therapist builds the patient’s capacity to bear the full extent of her feelings without becoming dysregulated by anxiety.

### Defensive Affects Restructuring

A second form of emotion dysregulation occurs when the patient’s defenses cause a defensive affect (Davanloo, 1990, 2000; Coughlin della Selva, 1996; Frederickson, 2013; Abbass, 2015). For example, a woman is assaulted by a man (stimulus in reality). This triggers anger (“true” feeling) and, as a result, she can fight him off. However, suppose she shows up in a therapist’s office and is terrified of the therapist, imagining that he is angry. Here she projects her anger upon the therapist (defense). Her defense of projection creates the defensive affect of fear. When the patient’s fear results from projection, the therapists need to deactivate the projection (Beck and Emery, 1985; Davanloo, 2000; Frederickson, 2013). Then, anxiety resulting from the projection will drop. Here, cognitive and experiential dynamic therapists agree. Let’s assume the therapist asked what feelings the patient had toward an assailant and she replied, “I’m afraid you might criticize me” (Defense of projection). Then she becomes weepy and depressed (Defensive affect). First, the therapist will help the patient see the defense of projection and then help her see the costs to increase her motivation of not using it anymore. If the patient stops relying on defenses, anxiety and defensive affects drop.

### Emotional Recognition, Expression, and Experiencing

To prevent future relapse, experiential dynamic therapists will also explore the true feeling which triggered the defense (Davanloo, 1990, 2000; Coughlin della Selva, 1996; Frederickson, 2013). The therapist can explore the feeling underneath the defense of self-criticism. By exploring the patient’s feelings, the therapist helps the patient feel her feelings without using defenses which formerly caused dysregulating emotions. By doing so, the therapist builds the patient’s capacity for tolerating high levels of affect without using a dysregulating defense (Davanloo, 1990, 2000; Coughlin della Selva, 1996; Greenberg and Vandekerckhove, 2008; Frederickson, 2013; Abbass, 2015). Now she can channel her “true” feelings into adaptive action with less risk of relapse (Johansson et al., 2014). See Table 3.

**TABLE 3:** Experiential-dynamic techniques for regulating emotions.

Mode	Regulatory process (according to EDER)	Strategy/Intervention	Model of therapy
Experiential-Dynamic	Anxiety regulation	– Identification – Enhancing bodily awareness – Differentiating feeling from anxiety – Introducing isolation of affect – Changing the pathway of unconscious anxiety discharge	ISTDP, AEDP, APT, IE-DP
Experiential-Dynamic	Defensive affects restructuring	– Blocking the defense – Identifying the defense – Clarifying the price of the defense – Clarifying the function of the defense – Pointing out causality, – Differentiating reality from fantasy – Changing the system of defenses used by the patient	ISTDP, AEDP, APT, IE-DP
Experiential-Dynamic	Emotion recognition	– Identification – Labeling – Enhancing bodily awareness – Helping to observe emotions – Differentiating feelings from anxiety and defenses – Differentiating true feelings from defensive affects	ISTDP, AEDP, APT, IE-DP
Experiential-Dynamic	Emotion expression	– Experiencing feeling physically in the body – Experiencing the impulse physically in the body – Building affect tolerance – Encourage *in vivo* desensitization	ISTDP, AEDP, APT, IE-DP
Relational	Emotion experiencing	– Focusing and facilitating patient-therapist interactions and explore the generated affects – Validating, affirming, encouraging patient’s affective experience – Expression of therapist’s empathic and affective response – Monitoring, mirroring and make explicit non-verbal responses	AEDP

*EDER, experiential-dynamic emotion regulation; ISTDP, intensive short term dynamic psychotherapy; AEDP, accelerated empathic dynamic psychotherapy; APT, affect phobia therapy; IE-DP, intensive experiential dynamic psychotherapy.*

## Cognitive-Experiential Techniques

Experiential techniques have been originally proposed in the context of Gestalt therapy (Perls et al., 1951), Experiential-dynamic therapies (Davanloo, 1990), and Transactional Analysis (Erskine, 2010). However, they have been incorporated in recent models of cognitive therapy such as Schema Therapy (Young et al., 2003), and partially also into Acceptance and Commitment therapy (Hayes et al., 2011). In this section we focus on Schema Therapy as an example of this integration of cognitive and experiential strategies to treat emotion regulation problems. Indeed, beside cognitive and behavioral interventions, Schema Therapy (ST) uses experiential techniques to target emotion dysregulation. Schema Therapy proposes that Early Maladaptive Schemas (EMS) and pathological Modes underlie and cause emotional dysregulation (Young et al., 2003; Kellogg and Young, 2006; Lobbestael and Arntz, 2010; Dadomo et al., 2016, 2018; Grecucci et al., 2018). We understand emotion dysregulation by focusing on four different macro-categories of modes (Dadomo et al., 2016, 2018). These modes are associated with specific dysregulated emotions. Innate Child Modes (that include childhood states posited as innate such as Vulnerable, Angry, Impulsive, and Contented Child Modes), dysfunctional Coping Modes (that include Compliant, Detached Protector, and Overcompensating Modes), dysfunctional Parent Modes (Punitive and Demanding/Critical Modes) and an overall functional Healthy Adult Mode.

Within ST, the techniques that address EMS draw on the canon of CBT techniques to correct for the distortions in beliefs about self and others. Dysregulated emotion is therefore achieved by addressing negative schemas by using the CER model as outlined above in our discussion on CBT. However, ST introduces an additional experiential dimension to shift negative schemas and regulate emotion by using “Modes.” Mode work proceeds by (1) identifying the operative Mode, (2) deactivating or reducing the intensity of the dysfunctional Mode, and (3) activating a more functional Mode.

The patient’s specific interpersonal difficulties are discussed to help them access and elaborate negative emotions related to particular modes (Kellogg and Young, 2006). The ST therapist monitors the patient’s type of emotions and manner of their expression to help the patient identify which Mode may be active (e.g., the patient who avoids feeling during the recollection of trauma is activating the dysfunctional Coping Mode of *Detached Protector*).

### Limited Reparenting

At the core of the ST approach is the therapist’s use of *Limited Reparenting* that provides a corrective emotional-relational experience, and produces a great source of interpersonal emotion regulation. The therapist responds to the patient’s developmental and attachment needs (Arntz, 2011) by providing warmth, empathy, compassion, as well as showing firmness, reciprocity, a respect for limits, and a recognition of the patient’s rights (Dadomo et al., 2016). Such limited reparenting seeks to develop the patient’s *Healthy Adult* Mode, which is a position from which the patient can see their own needs, understand reality, and become more autonomous. The patient thus internalizes the therapist’s modeling of healthy adult function and emotional regulation abilities. The Critical Parent Mode has to be overcome before the patient can allow themselves within their Child Mode to experience and express feeling and desires that were banished or frustrated by the dysfunctional Parent Modes.

### Emphatic Confrontation

Experiential techniques include *empathic confrontation, imagery rescripting*, and *chair work.* After deactivating the dysfunctional mode, a more functional one is activated: for example, the (*Functional) Happy Child* (Young et al., 2003; Martin and Young, 2010). In this step, the patient experiences an up-regulation of positive emotions, but also self-soothing, calming emotions that down-regulate unpleasant emotions. One of the key elements in all ST’s techniques is the “validation process.” What the patient feels is never “wrong,” and all emotions do have a meaning. This validation of dysfunctional modes of coping is reached through *Empathic confrontation*. During sessions, the therapist shows that he/her understands why the patient shows dysfunctional patterns, and explains how they are relative to traumatic childhood experiences Beyond empathic confrontation, an empathic attitude of the therapist can be considered the basis for all therapeutic interventions. Communication research has largely contributed to the description of empathic interventions emphasizing the role of non-verbal aspects such as prosody or physiological synchronization (Wynn and Wynn, 2006; Cameron, 2011; Messina et al., 2013; Buchholz, 2014; Weiste and Peräkylä, 2014; Peräkylä et al., 2015; Buchholz et al., 2017; Voutilainen et al., 2018).

### Imagery Rescripting

When the patient is emotionally highly activated during a session, and shows his/her vulnerability, *imagery rescripting* exercises are used, in which schemas and modes are activated with their associated unpleasant emotions (Holmes et al., 2007; Arntz, 2012). Imagery rescripting represent a mean to regulate traumatic dysregulated emotions of the past. The goal of these exercises is to rewrite traumatic childhood experiences and help the patient elaborate dysregulated emotions, giving them new meaning through the therapist’s intervention in imagery (Arntz, 2011).

### Chair-Work

Patients that show strong emotional dysregulation often show low metacognition levels: they struggle to understand what happens inside them when triggers are activated. This situation causes fast flipping from one mode to another, in what the patients describe as a strong internal confusion. This is often associated to a strong feeling of discomfort. For this reason, *chair-work* is used in ST (Kellogg and Young, 2006). This technique is based on the dialogue between the patient’s different modes, so that he/she can understand which mode is active and the dysregulation it produces (Van Vreeswijk et al., 2015). Some modes, such as *Vulnerable Child* and *Dysfunctional Parent*, are closely integrated. These modes are correlated to the patient’s feeling of guilt, vulnerability and sadness. The patient gains awareness through chair-work on how guilt is caused by a *Dysfunctional Parent*, an internalization of past interactions with his/her caregivers that causes sadness and anguish to the *Vulnerable Child*. Learning to distinguish different modes is the basis for functionally coping with them (Ball, 1998; Holmes et al., 2007; Edwards and Arntz, 2012). In the last years experiential exercises are found to be the most efficient in handling emotional dysregulation and changing EMSs. The emotional techniques used in Schema Therapy (ST) are been hypothesized to be able to reach emotional structures of the brain such as the amygdala, in which emotional memories are stored (LeDoux et al., 1989). In ST, the therapist helps the patient activate these memories, going from present emotion and body sensation to the very past ones, to satisfy unmet needs (Martin and Young, 2010). Emotional memories are then rewritten through experiential techniques, such as partial reparenting and imagery rescripting. In this way, ST fosters emotion regulation (Table 4).

**TABLE 4:** Cognitive-experiential techniques for regulating emotions.

Mode	Regulatory process	Strategy/Intervention	Model of therapy
Relational	Appraisal, response modulation	Limited reparenting: – Understanding the basic emotional needs not met in patient’s life – Establishing a secure attachment with the therapist – The therapist meets these needs within the bounds of a professional relationship	ST
Relational	Attention, appraisal, response modulation	Emphatic confrontation: – Identifying the coping mode in the session – Validation of coping mode – Emphatic understanding of basic needs and emotions – Memory of emotional needs not satisfied in life events – Showing our intention to do something to protect the vulnerable part in a functional way	ST
Experiential	Attention, appraisal, response modulation	Imagery rescripting: – Creating a safe place – Recognizing an actual specific need not met in a present situation – Creating a bridge with past situations and meeting unfulfilled needs using reparenting – Understanding the continuity of needs – Focus on the specific emotion felt during and after reparenting – Understanding how to meet basic emotional needs – Coming back to the present and feeling the difference – Being aware about the link between past traumatic experience and present feelings – Building a healthy internal working model	ST
Emotional focused	Attention, appraisal, response modulation	Chair works: – Creating a link between the modes and chair – Ability to recognize ad differentiate different mental states – Moving from one chair to another by active mode at that time – Awareness about the internal dynamics	ST/Gestalt

## Two Biological Mechanisms Behind Emotion Regulation in Psychotherapy and an Integrative Proposal

Scholars agree on the necessity of incorporating findings and strategies from different therapeutic models into their clinical practice. In this paper we put forward the idea that the clinician can integrate a wide range of techniques to foster emotion regulation according to the needs of their patients. One strategy can work for one patient, but not for the another one. In paragraph 2, 3, and 4, we presented briefly different sets of approaches to reach this aim. In this paragraph, we suggest a dual route model of emotion regulation that integrates the two approaches described above (CER and EDER) and that can guide the clinician to select the best strategy to foster a better emotional experience in his/her patients. We believe CER and EDER as two complementary approaches that rely on parallel pathways that enable emotion regulation in patients. CER is a more cognitive-attentive and top-down process, EDER is a more emotion focused and bottom-up way of reworking dysregulated affective states. This division parallels previous considerations of other authors regarding “changing emotions with cognition” vs. “changing emotion with emotion” (Greenberg, 2004) and other models of dual route in psychotherapy (see for example, Levin, 2010).

We make the point that these two approaches rely on two different psychological and neural mechanisms: memory extinction and reconsolidation. Until 1989 neuroscientists agreed on the fact that once an (emotional) learning had occurred, it was permanently stored in memory circuits (LeDoux et al., 1989; Roozendaal et al., 2009). The only possibility to “change” that learning was to suppress it temporally with a new learning. A procedure known as Extinction, that will briefly outline here. One way to study the formation of fear memories is Pavlovian conditioning. During Pavlovian conditioning, a neutral stimulus (e.g., a tone, Conditioned stimulus, CS), is paired with an aversive stimulus (e.g., footshock, Unconditioned stimulus, US). The US elicits spontaneous fear reactions (freezing, avoidance, etc.). After a number of tone-footshock pairings, the CS becomes able to elicit fear reactions (Conditioned response, CR). Now a new learning has been acquired (say memory trace #1). Extinction refers to a decrease in the emission of CR when CS is presented due to a formation of a new learning (memory trace #2 due to CS repeatedly presented without the US). In other words, when a conditioned stimulus is presented alone, and no longer predicts the coming of the unconditioned stimulus, CR gradually stops. In psychotherapy, extinction occurs when the patient is re-exposed to the conditioned cue or conditioned context (say for example, an elevator where the panic attacks started), in the absence of the unconditioned stimulus (panic attack). In neural terms, the memory trace #1 is not erased nor replaced by memory trace #1. The proof of this is that in certain situations the memory trace #1 spontaneously becomes reactivated (CR following CS). We hypothesize that CER and the family of standard Cognitive-Behavioral therapies, mainly rely on Extinction. The therapist helps the patient to counteract past learning (in the form of beliefs, attentive mechanisms, behavioral responses, etc.) by adding new learning (positive thinking, attention training, exposure…).

**FIGURE 1:**
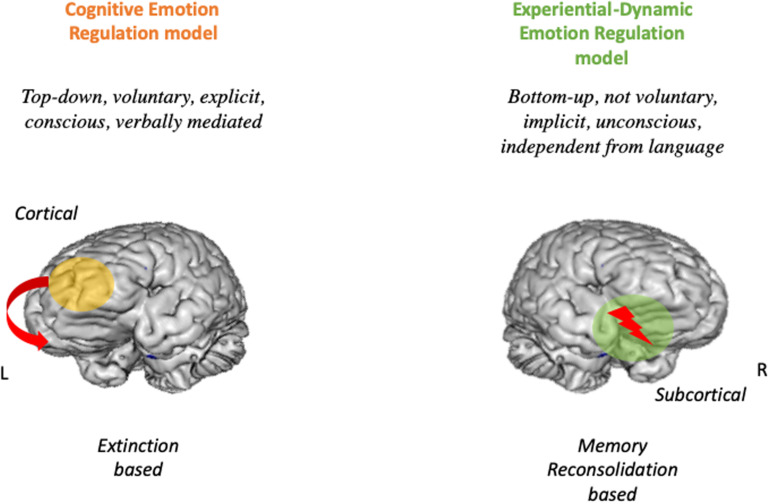
The dual route model of emotion regulation. On the Left the Cogntive emotion regulation route. ON the Right the Experiential dynamic emotion regulation model.

In this paper, we have presented some examples of cognitive based techniques that may rely on this biological process (see section “Experiential-Dynamic Techniques to Regulate Emotions”). Although this is undoubtedly helpful for the patient and proved to work, one problem of extinction-based techniques, is that similarly to other kind of re-learnings, they are subject to relapse in time (CR following CS). The indelibility of the memory traced, implied strong limits to the possibility of effective transformations in psychotherapy (Ecker et al., 2013). The only possibility was a long relearning. However, from 1997 the panorama dramatically changed and new challenging evidence of the possibility to complete erase previous learned emotional learning was provided (Sekiguchi et al., 1997; Roullet and Sara, 1998; Przybyslawski et al., 1999; Nader et al., 2000; Nader and Einarsson, 2010; Ecker et al., 2013). Contrary to previous “static” models of memory, the new vision of a “dynamic memory” states that every time a memory is retrieved, the memory trace is labile and can incorporate new learning that potentially alters and even erase the original trace. Starting from animal experiments, neuroscientists showed that target emotional memories can be reactivated in a labile (plastic) state, that allows the learning to be nullified (Ecker et al., 2012). This process is known as Memory Reconsolidation (Nader et al., 2000; Nader and Einarsson, 2010) and has been tested in various laboratory settings and recently in more clinical settings (Ecker, 2006, 2008). For memory reconsolidation to happen, a series of steps must be followed. First, the target emotional learning must be reactivated. The memory trace is now in a labile state for a few hours (Nader et al., 2000; Nader and Einarsson, 2010). The fate of this memory trace depends on what happens in the following hours. It can be re-consolidated, if an analog experience happens, or de-consolidated. For de-consolidation to occur, a critical additional experience must be provided while the memory is in this labile state (Pedreira et al., 2004). This experience must contain new elements that create a mismatch with the previous learning (Ecker et al., 2012). When this happens, the emotional memory is erased and the mechanisms elicited by that learning are no longer to elicited. Notably, the autobiographical memory of the event that originally produced the emotional learning is not impaired at all (Debiec et al., 2006). What is erased is the emotional impact of that event and the consequences that it brings (symptoms). We hypothesize that once a Memory Reconsolidation process is reached in the therapeutic settings, the dysregulated affect and the associated mechanisms that produce the dysregulation stop. Interestingly, different models of therapies (almost belonging to experiential approaches, but not restricted to), in the recent past arrived at similar conclusions and implemented similar processes in their practice even before Memory Reconsolidation was discovered (see for example, Accelerated Experiential Dynamic Therapy, Fosha, 2000; Coherence Therapy, Ecker et al., 2012; ISTDP, Davanloo, 1990, 2000; Emotion-Focused Therapy, Greenberg, 2004; Psychoanalytic Therapy, Gorman and Roose, 2011, and others). We believe all the treatment modalities based on active working and reworking of target emotional learnings (by means of experiential techniques), foster Memory Reconsolidation. In this paper, we have presented some examples of experiential therapies that may foster this biological curative process (see section “Cognitive-Experiential Techniques”). See Figure 1.

## Conclusion

To sum up, in this paper we presented two approaches and related techniques to foster emotion regulation in patients. We believe both approaches can be useful and provide a wide plethora of techniques that can enrich the toolbox of every therapist. On one hand, CER approach considers emotions as generated by appraisal and attentive processes. The clinician helps the patient to reach better regulation by teaching new regulatory strategies at the thinking level (mainly modifying pathological appraisals), or at an attentional level (i.e., selective attention for negative cues), or at a behavioral level (exposure, etc.). Memory extinction may be the core biological mechanism behind CBT techniques. The therapist helps the patient to activate top-down mechanism (mainly situated in the prefrontal cortex), to modulate subcortical areas responsible for emotion activation (amygdala, insula, etc.). Consistently with this proposal, studies of neurobiological changes due to CBT have shown increased activation in the frontal cortical regions, such as prefrontal cortex and anterior cingulate cortex (i.e., top-down processing), and decreased activity in subcortical regions. Just to cite some of these studies, augmented prefrontal activity has been found for panic disorder (Prasko et al., 2004), depression (Goldapple et al., 2004) and many others (see Beauregard, 2014 for a review) after therapy.

On the other hand, EDER approach (Frederickson et al., 2018), focuses on emotion itself, and not to cognitive, attentive or behavioral mechanisms to change emotional responses. As said before, EDER considers emotion and normal emotion regulation as biological process independent from cognition and conscious effort. EDER consider dysregulation as generated by additional dysregulatory mechanisms (conditioned anxiety and defensive mechanisms created by unpleasant life experiences). The therapist helps the patient to remove these mechanisms by asking the patient to attend and fully experience warded off emotions without covering them with anxiety and defensive mechanisms. In other words, by adding a new opposite experience (say for example the experience of anger toward a dismissing mother, instead of anxiety and/or defensive affects that covered that anger), memory reconsolidation in subcortical areas (supposingly, the amygdala and other key regions), but also in fronto-ventral areas (orbito-frontal cortex, ventromedial PFC, and ventral cingulate cortex, Gyurak and Etkin, 2014) is thus facilitated. Evidence of the involvement of memory reconsolidation during Experiential-Dynamic therapies are not available yet. Further research is necessary to test this hypothesis.

We now make the point that therapists can use both sets of techniques (and activate both neural mechanisms) at their convenience. In this way the therapist can take advantage of both sets of techniques and adapt to the needs and specific regulation problems of her/his patient. Further research is needed to better understand the ways both methodologies can be integrated and when this is the case.

In conclusion, in this paper we pointed out that there are two main modalities (CER vs. EDER) and descending groups of techniques (mainly cognitive vs. mainly experiential) to help the patient in better regulating distressing emotions. Both routes serve the function to foster emotion regulation in patients and can be integrated in clinical practice. Future research will explore in further details differences and commonalities of these two classes of processes and how they might influence emotion regulation problems. The clinician may want to select one or the other modality to help their patients to achieve better regulation of emotions. It’s time for such an integration.

## Author Contributions

AG wrote the main concepts and specific sections and supervised the whole process of writing. JF, IM, CC, HD, MP, and AT wrote the specific sections of the manuscript. AG, LA, and GL revised and finalized the final version of the manuscript.

## Conflict of Interest

The authors declare that the research was conducted in the absence of any commercial or financial relationships that could be construed as a potential conflict of interest.

## References

[B1] Abbass A. (2015). *Reaching through the Resistance. Advanced Psychotherapy Techniques.* Kansas City: Seven Leaves Press.

[B2] Abramowitz J. S. Tolin D. F. Street G. P. (2001). Paradoxical effects of thought suppression: a meta-analysis of controlled studies. *Clin. Psychol. Rev.* 21 683–703. 11434226 10.1016/s0272-7358(00)00057-x

[B3] Arntz A. (2011). Imagery rescripting for personality disorders. *Cogn. Behav. Pract.* 18 466–481.

[B4] Arntz A. (2012). Imagery rescripting as a therapeutic technique: review of clinical trials, basic studies, and research agenda. *J. Exp. Psychopathol.* 3 189–208.

[B5] Ball S. A. (1998). Manualized treatment for substance abusers with personality disorders: dual focus schema therapy. *Addict. Behav.* 23 883–891. 9801723 10.1016/s0306-4603(98)00067-7

[B6] Beauregard M. (2014). Functional neuroimaging studies of the effects of psychotherapy. *Dialog. Clin. Neurosci.* 16 75–81. 10.31887/DCNS.2014.16.1/mbeauregard PMC3984893 24733972

[B7] Beck A. Emery G. (1985). *Anxiety Disorders and Phobias: A Cognitive Perspective.* New York, NY: Basic Books.

[B8] Beck J. S. (2011). *Cognitive Behavior Therapy: Basics and Beyond (2e).* New York, NY: Guilford Press.

[B9] Benjamin L. (1993). *Interpersonal diagnosis and treatment of personality disorders.* New York, NY: Guilford Press.

[B10] Berne E. (1961). *Transactional Analysis in Psychotherapy: A Systematic Individual and Social Psychiatry.* London: Souvenir Press.

[B11] Bowlby J. (1969). *Attachment and Loss, vol. 1: Attachment.* New York, NY: Basic Books.

[B12] Bowlby J. (1973). *Attachment and Loss, vol. 2: Separation Anxiety and Anger.* New York, NY: Basic Books.

[B13] Bowlby J. (1980). *Attachment and Loss, vol.3: Loss Sadness and Depression.* New York, NY: Basic Books.

[B14] Buchholz M. B. (2014). Patterns of empathy as embodied practice in clinical conversation - a musical dimension. *Front. Psychol.* 5:349. 10.3389/fpsyg.2014.00349 24817855 PMC4013836

[B15] Buchholz M. B. Bergmann J. R. Alder M.-L. Dittmann M. M. Dreyer F. Kächele H. (2017). “The building of empathy: conceptual ‘pillars’ and conversational practices in psychotherapy,” in *Empathy - An Evidence-Based Interdisciplinary Perspective*, ed. Kondo M. (London: InTech), 10.5772/intechopen.69628

[B16] Cameron L. (2011). *Metaphor and Reconciliation: The Discourse Dynamics of Empathy In Post-Conflict Conversations.* New York, NY: Routledge.

[B17] Campos J. J. Frankel C. B. Camras L. (2004). On the nature of emotion regulation. *Child. Dev.* 75 377–394. 15056194 10.1111/j.1467-8624.2004.00681.x

[B18] Coughlin della Selva P. (1996). *Intensive Short-Term Dynamic Psychotherapy. Theory and Practice.* New York, NY: John Wiley & Sons.

[B19] Dadomo H. Grecucci A. Giardini I. Ugolini E. Carmelita A. Panzeri M. (2016). Schema therapy for emotional dysregulation: theoretical implication and clinical application. *Front. Psychol.* 7:1987 10.3389/fpsyg.2016.01987 PMC5177643 28066304

[B20] Dadomo H. Panzeri M. Caponcello D. Carmelita A. Grecucci A. (2018). Schema therapy for emotional dysregulation in personality disorders: a review. *Curr. Opin. Psychiatry* 31 43–49. 10.1097/YCO.0000000000000380 29120915

[B21] Damasio A. (1999). *The Feeling of What Happens: Body and Emotion in the Making Of Consciousness.* San Diego, CA: Harcourt College Publishers.

[B22] Davanloo H. (1990). *Unlocking the Unconscious.* Chichester: John Wiley & Sons.

[B23] Davanloo H. (2000). *Intensive Short-Term Dynamic Psychotherapy: Selected Papers of Habib Davanloo.* Chichester: John Wiley & Sons.

[B24] De Panfilis C. Schito G. Generali I. Gozzi L. Ossola P. Marchesi C. (2019). Emotions at the border: increased punishment behavior during fair interpersonal exchanges in Borderline Personality Disorder. *J. Abnorm. Psychol.* 128 162–172. 10.1037/abn0000404 30714797

[B25] Debiec J. Doyère V. Nader K. LeDoux J. E. (2006). Directly reactivated, but not indirectly reactivated, memories undergo reconsolidation in the amygdala. *Proc. Natl. Acad. Sci. U.S.A.* 103 3428–3433. 16492789 10.1073/pnas.0507168103 PMC1413871

[B26] Ecker B. (2006). “The effectiveness of psychotherapy,” in *Keynote address, 12th Biennial Conference of the Constructivist Psychology Network* (San Marcos, CA: University of California).

[B27] Ecker B. (2008). Unlocking the emotional brain: Finding the neural key to transformation. *Psychother. Netw.* 32:60.

[B28] Ecker B. Ticic R. Hulley L. (2012). *Unlocking the Emotional Brain: Eliminating Symptoms at Their Roots Using Memory Reconsolidation.* Abingdon: Routledge.

[B29] Ecker B. Ticic R. Hulley L. (2013). A primer on memory reconsolidation and its psychotherapeutic use as a core process of profound change. *Neuropsychotherapist* 1 82–99.

[B30] Edwards D. Arntz A. (2012). “Schema therapy in historical perspective,” in *The Wiley-Blackwell Handbook of Schema Therapy: Theory, Research, and Practice*, eds van Vreeswijk M. Broersen J. (Hoboken, NJ: John Wiley & Sons), 3–26.

[B31] Erskine R. G. (2010). Integrating expressive methods in a relational-psychotherapy. *Int. J. Integr. Psychother.* 1

[B32] Fosha D. (2000). *The Transforming Power of Affect: A Model For Accelerated Change.* New York, NY: Basic Books.

[B33] Frederickson J. (2013). *Co-Creating Change: Effective Dynamic Therapy Techniques.* Kansas City, MO: Seven Leaves Press.

[B34] Frederickson J. Messina I. Grecucci A. (2018). Dysregulated affects and dysregulating defenses: toward an emotion regulation informed dynamic psychotherapy. *Front. Psychol.* 9:2054. 10.3389/fpsyg.2018.02054 30455650 PMC6230578

[B35] Frijda N. (1986). *The Emotions.* Cambridge: Cambridge University Press.

[B36] Gabbard G. O. (2014). *Psychodynamic Psychiatry in Clinical Practice.* Washington, DC: American Psychiatric Press.

[B37] Gilbert P. (2009). Introducing compassion-focused therapy. *Adv. Psychiatr. Treat.* 15 199–208.

[B38] Giorgetta C. Grecucci A. Rattin A. Guerreschi C. Sanfey A. Bonini N. (2014). To play or not to play: A personal dilemma in pathological gambling. *Psychiatry Res.* 219 562–569. 10.1016/j.psychres.2014.06.042 25024055

[B39] Giorgetta C. Grecucci A. Zanon S. Perini L. Balestrieri M. Bonini N. (2012). Reduced risk-taking behaviour as a trait feature of anxiety. *Emotion* 12 1373–1383. 10.1037/a0029119 22775123

[B40] Goldapple K. Segal Z. Garson C. Lau M. Bieling P. Kennedy S. (2004). Modulation of cortical-limbic pathways in major depression: treatment-specific effects of cognitive behavior therapy. *Arch. Gen. Psychiatry* 61 34–41. 14706942 10.1001/archpsyc.61.1.34

[B41] Gorman J. M. Roose S. P. (2011). The neurobiology of fear memory reconsolidation and psychoanalytic theory. *J. Am. Psychoanal. Assoc.* 59 1201–1219. 10.1177/0003065111427724 22080504

[B42] Grecucci A. (2012). *Il Conflitto Epistemologico. Psicoanalisi e Neuroscienze Dei Processi Anticonoscitivi.* Italy: Edizioni Psiconline.

[B43] Grecucci A. Chiffi D. Di Marzio F. Frederickson J. Job R. (2016). “Anxiety and its regulation: Neural mechanisms and regulation techniques according to the Experiential-Dynamic approach,” in *New Developments in Anxiety Disorders*, eds Durbano F. Marchesi B. (InTech Publishing).

[B44] Grecucci A. De Pisapia N. Kusalagnana Thero D. Paladino M. P. Venuti P. Job R. (2015a). Baseline and strategic effects behind mindful emotion regulation. Behavioral and physiological investigation. *PLoS One*:e116541. 10.1371/journal.pone.0116541 25590627 PMC4295876

[B45] Grecucci A. Theuninck A. Frederickson J. Job R. (2015b). “Mechanisms of social emotion regulation: from neuroscience to psychotherapy,” in *Emotion Regulation: Processes, Cognitive Effects and Social Consequences*, ed Bryant M. L. (New York, NY: Nova Publishing), 57–84.

[B46] Grecucci A. Giorgetta C. Bonini N. Sanfey A. G. (2013a). Living emotions, avoiding emotions: behavioral investigation of the regulation of socially driven emotions. *Front. Psychol.* 3:2013. 10.3389/fpsyg.2012.00616 23349645 PMC3552385

[B47] Grecucci A. Giorgetta C. Bonini N. Sanfey A. G. (2013b). Reappraising social emotions: the role of inferior frontal gyrus, temporo-parietal junction and insula in interpersonal regulation”. *Front. Hum. Neurosci.* 7:e523. 10.3389/fnhum.2013.00523 24027512 PMC3759791

[B48] Grecucci A. Giorgetta C. van Wout M. Bonini N. Sanfey A. G. (2013c). Reappraising the Ultimatum: an fMRI study of emotion regulation and decision-making. *Cereb. Cortex* 23 399–410. 22368088 10.1093/cercor/bhs028

[B49] Grecucci A. Job R. (2015). Rethinking reappraisal: Insights from Affective Neuroscience. *Behav. Brain Sci.* 38:e102. 10.1017/S0140525X14001538 26786023

[B50] Grecucci A. Job R. Frederickson J. (eds) (2017). Advances in emotion regulation: from neuroscience to psychotherapy. *Front. Psychol.* 8:985 10.3389/fpsyg.2017.00985 PMC5479113 28680409

[B51] Grecucci A. Messina I. Dadomo A. (2018). Decoupling internalized dysfunctional attachments: a combined ACT and schema therapy approach. *Front. Psychol.* 9:2332 10.3389/fpsyg.2018.02332 PMC6265412 30532729

[B52] Grecucci A. Sulpizio S. Vespignani F. Job R. (2019). Seeing emotions, reading emotions: behavioral and ERPs evidence of the regulation of visual and linguistic stimuli. *PLoS One*:e0209461. 10.1371/journal.pone.0209461 PMC6544208 31150397

[B53] Greenberg L. S. (2004). Emotion–focused therapy. *Clin. Psychol. Psychother.* 11 3–16. 10.1002/cpp.624 19639649

[B54] Greenberg L. S. Bolger E. (2001). An emotion-focused approach to the overregulation of emotion and emotional pain. *J. Clin. Psychol.* 57 197–211. 11180147 10.1002/1097-4679(200102)57:2<197::aid-jclp6>3.0.co;2-o

[B55] Greenberg L. S. Vandekerckhove M. (2008). “Emotional expression and regulation in psychotherapeutic processes,” in *Regulating Emotions: Social Necessity and Biological Inheritance*, eds Vandekerckhove M. Scheve C. V. Ismer S. Kronast S. (New York, NJ: Blackwell Press), 240–268.

[B56] Greenberger D. Padesky C. A. (2015). *Mind Over Mood: Change How You Feel by Changing the Way You Think.* New York, NY: Guilford Press.

[B57] Gross J. J. (1998). The emerging field of emotion regulation: an integrative review. *Rev. Gen. Psychol.* 2 271–299.

[B58] Gross J. J. (ed.) (2014). *Handbook of Emotion Regulation*, 2nd Edn New York, NY: Guilford Press.

[B59] Gyurak A. Etkin A. (2014). “A neurobiological model of implicit and explicit emotion regulation,” in *Handbook of Emotion Regulation, 2nd edn*, ed. gross J. J. (New York, NY: Guilford Press), 76–90.

[B60] Hawton K. Salkovskis P. M. Kirk J. Clark D. M. (1989). *Cognitive Behaviour Therapy for Psychiatric Problems: A practical Guide.* Oxford: Oxford University Press.

[B61] Hayes S. C. Storsahl K. D. Wilson K. G. (2011). *Acceptance and Commitment Therapy: The Process and Practice of Mindful Change (2e).* London: Guilford Press.

[B62] Herwig U. Kaffenberger T. Jäncke L. Brühl A. B. (2010). Self-related awareness and emotion regulation. *Neuroimage* 50 734–741. 10.1016/j.neuroimage.2009.12.089 20045475

[B63] Holmes E. A. Arntz A. Smucker M. R. (2007). Imagery rescripting in cognitive behaviour therapy: images, treatment techniques and outcomes. *J. Behav. Ther. Exp. Psychiatry* 38 297–305. 18035331 10.1016/j.jbtep.2007.10.007

[B64] Hunt M. G. (1998). The only way out is through: emotional processing and recovery after a depressing life event. *Behav. Res. Ther.* 36 361–384. 9670599 10.1016/s0005-7967(98)00017-5

[B65] Johansson R. Town J. Abbass A. (2014). Davanloo’s Intensive Short-Term Dynamic Psychotherapy in a tertiary psychotherapy service: overall effectiveness and association between unlocking the unconscious and outcome. *Peer J.* 2:e548. 10.7717/peerj.548 PMC4157301 25210661

[B66] Kellogg S. H. Young J. E. (2006). Schema therapy for borderline personality disorder. *J. Clin. Psychol.* 62 445–458. 16470629 10.1002/jclp.20240

[B67] Kring A. M. Werner K. H. (2004). “Emotion regulation and psychopathology,” in *The Regulation Of Emotion*, eds Philippot P. Feldman R. S. (Hove: Psychology Press), 359–385.

[B68] Kuyken W. Padesky C. A. (2011). *Collaborative Case Conceptualization: Working Effectively with Clients in Cognitive-Behavioral Therapy.* New York, NY: Guilford Press.

[B69] LeDoux J. E. Romanski L. Xagoraris A. (1989). Indelibility of subcortical emotional memories. *J. Cogn. Neurosci.* 1 238–243. 10.1162/jocn.1989.1.3.238 23968507

[B70] Levin P. A. (2010). *In an Unspoken Voice: How the Body Releases Trauma and Restores Goodness.* Berkeley, CA: North Atlantic Books.

[B71] Linehan M. (1993a). *Cognitive-Behavioral Treatment of Borderline Personality Disorder.* New York, NY: Guilford Press.

[B72] Linehan M. (1993b). *Skills Training Manual for Treating Borderline Personality Disorder.* New York, NY: Guilford Press.

[B73] Lobbestael J. Arntz A. (2010). Emotional, cognitive and physiological correlates of abuse-related stress in borderline and antisocial personality disorder. *Behav. Res. Ther.* 48 116–124. 10.1016/j.brat.2009.09.015 19854433

[B74] Lutz A. McFarlin D. R. Perlman D. M. Salomons T. V. Davidson R. J. (2012). Altered anterior insula activation during anticipation and experience of painful stimuli in expert meditators. *Neuroimage* 64 538–546. 10.1016/j.neuroimage.2012.09.030 23000783 PMC3787201

[B75] Martin R. Young J. (2010). “Schema therapy,” in *Handbook of Cognitive-Behavioral Therapies*, Ed. Dobson K. S. (New York, NY: Guilford Publications), 317.

[B76] McCullough L. (1997). *Changing Character: Short-term Anxiety-regulating Psychotherapy For Restructuring Defenses, Affects, And Attachment.* New York, NY: Basic Books.

[B77] Messina I. Bianco F. Cusinato M. Calvo V. Sambin M. (2016). Abnormal default system functioning in depression: implications for emotion regulation. *Front. Psychol.* 7:858. 10.3389/fpsyg.2016.00858 27375536 PMC4901076

[B78] Messina I. Grecucci A. Marogna C. Calvo V. (2020). Relational exposure as mechanisms of change in psychodynamic psychotherapy: convergences between psychotherapy research and affective neuroscience. *TPM* 27 1–14.

[B79] Messina I. Palmieri A. Sambin M. Kleinbub J. R. Voci A. Calvo V. (2013). Somatic underpinnings of perceived empathy: the importance of psychotherapy training. *Psychother. Res.* 23 169–177. 10.1080/10503307.2012.748940 23234457

[B80] Nader K. Einarsson E. O. (2010). Memory reconsolidation: an update. *Ann. N. Y. Acad. Sci.* 1191 27–41. 10.1111/j.1749-6632.2010.05443.x 20392274

[B81] Nader K. Schafe G. E. LeDoux J. E. (2000). Fear memories require protein synthesis in the amygdala for reconsolidation after retrieval. *Nature* 406 722–726. 10963596 10.1038/35021052

[B82] Ochsner K. N. Bunge S. A. Gross J. J. Gabrieli J. D. (2002). Rethinking feelings: an fMRI study of the cognitive regulation of emotion. *J. Cogn. Neurosci.* 14 1215–1229. 12495527 10.1162/089892902760807212

[B83] Ochsner K. N. Gross J. J. (2005). The cognitive control of emotion. *Trends Cogn. Sci.* 9 242–249. 15866151 10.1016/j.tics.2005.03.010

[B84] Ochsner K. N. Gross J. J. (2008). Cognitive emotion regulation: insights from social cognitive and affective neuroscience. *Curr. Direct. Psychol. Sci.* 17 153–158. 10.1111/j.1467-8721.2008.00566.x PMC4241349 25425765

[B85] Opialla S. Lutz J. Scherpiet S. Hittmeyer A. Jäncke L. Rufer M. (2015). Neural circuits of emotion regulation: a comparison of mindfulness-based and cognitive reappraisal strategies. *Eur. Arch. Psychiatry Clin. Neurosci.* 265 45–55. 10.1007/s00406-014-0510-z 24902936

[B86] Osimo F. (2003). *Experiential Short-Term Dynamic Psychotherapy, a Manual, Bloomington.* Bloomington: Authorhouse.

[B87] Panksepp J. (1998). *Affective Neuroscience: the foundations of human and animal emotions.* Oxford: Oxford University Press.

[B88] Panksepp J. Biven L. (2012). *The Archaeology of Mind: Neuroevolutionary Origins of Human Emotions.* New York, NY: W. W. Norton & Company.

[B89] Pappaianni E. De Pisapia N. Siugzdaite R. Crescentini C. Calcagnì A. Job R. (2019). Less is more: psychological and morphometric differences between low vs high reappraisers. *Cogn. Affect. Behav. Neurosci.* 20 128–140. 10.3758/s13415-019-00757-5 31858436 PMC7613187

[B90] Pedreira M. E. Pérez-Cuesta L. M. Maldonado H. (2004). Mismatch between what is expected and what actually occurs triggers memory reconsolidation or extinction. *Learn. Mem.* 11 579–585. 15466312 10.1101/lm.76904 PMC523076

[B91] Peräkylä A. Henttonen P. Voutilainen L. Kahri M. Stevanovic M. Sams M. (2015). Sharing the emotional load. Recipient affiliation calms down the storyteller. *Soc. Psychol. Q.* 78 301–323. 10.1177/0190272515611054

[B92] Perls F. Hefferline G. Goodman P. (1951). *Gestalt Therapy.* New York, NY: Julian Press.

[B93] Prasko J. Horácek J. Záleský R. Kopecek M. Novák T. Pasková B. (2004). The change of regional brain metabolism (18FDG PET) in panic disorder during the treatment with cognitive behavioral therapy or antidepressants. *Neuro Endocrinol. Lett.* 25 340–348. 15580167

[B94] Przybyslawski J. Roullet P. Sara S. J. (1999). Attenuation of emotional and nonemotional memories after their reactivation: Role of beta adrenergic receptors. *J. Neurosci.* 19 6623–6628. 10414990 10.1523/JNEUROSCI.19-15-06623.1999 PMC6782794

[B95] Rogers C. R. (1951). *Client-Centred Therapy: Its Current Practice, Implications, and Theory.* Boston, MA: Houghton Mifflin.

[B96] Roozendaal B. McEwen B. S. Chattarji S. (2009). Stress, memory and the amygdala. *Nat. Rev. Neurosci.* 10 423–433. 10.1038/nrn2651 19469026

[B97] Roullet P. Sara S. J. (1998). Consolidation of memory after its reactivation: Involvement of beta noradrenergic receptors in the late phase. *Neural Plast.* 6 63–68. 9920683 10.1155/NP.1998.63 PMC2565312

[B98] Scherer K. R. Shorr A. Johnstone T. (eds) (2001). *Appraisal Processes in Emotion: Theory, Methods, Research.* Canary, NC: Oxford University Press.

[B99] Segal Z. V. Williams J. M. G. Teasdale J. D. (2012). *Mindfulness-Based Cognitive Therapy for Depression (2e) (MBCT).* London: Guilford Press.

[B100] Sekiguchi T. Yamada A. Suzuki H. (1997). Reactivation-dependent changes in memory states in the terrestrial slug *Limax flavus*. *Learn. Mem.* 4 356–364. 10706372 10.1101/lm.4.4.356

[B101] Sheppes G. (2014). “Emotion regulation choice: theory and findings,” in *Handbook of Emotion Regulation*, ed. Gross J. J. (New York, NY: Guilford Publications), 126–139.

[B102] Sheppes G. Scheibe S. Suri G. Gross J. J. (2011). Emotion-regulation choice. *Psychol. Sci.* 22 1391–1396. 10.1177/0956797611418350 21960251

[B103] Sorella S. Lapomarda G. Messina I. Siugzdaite R. Job R. Grecucci A. (2019). Testing the expanded continuum hypothesis of schizophrenia and bipolar disorder. Neural and psychological evidence for shared and distinct mechanisms. *Neuroimage* 23:101854. 10.1016/j.nicl.2019.101854 PMC6529770 31121524

[B104] ten Have-de Labije J. Neborsky R. (2012). *Mastering Intensive Short-Term Dynamic Psychotherapy: A Roadmap to the Unconscious.* London: Karnac Books.

[B105] Van Vreeswijk M. Broersen J. Nadort M. (2015). *The Wiley-Blackwell Handbook of Schema Therapy: Theory, Research and Practice.* Hoboken, NJ: John Wiley & Sons.

[B106] Vandekerckhove M. Kestemont J. Weiss R. Schotte C. Exadaktylos V. Haex B. (2012). Experiential versus analytical emotion regulation and sleep: breaking the link between negative events and sleep disturbance. *Emotion* 12 1415–1421. 10.1037/a0028501 22775124

[B107] Vandekerckhove M. Wang Y. (2018). Emotion, emotion regulation and sleep: an intimate relationship. *Aims Neurosci.* 5 1–17. 10.3934/Neuroscience.2018.1.1 32341948 PMC7181893

[B108] Vandekerckhove M. Weiss R. Schotte C. Exadaktylos V. Verbraecken J. Haex B. (2011). The role of presleep negative emotion in sleep physiology. *Psychophysiology* 48 1738–1744. 10.1111/j.1469-8986.2011.01281.x 21895689

[B109] Voutilainen L. Henttonen P. Kahri M. Ravaja N. Sams M. Peräkylä A. (2018). Empathy, challenge, and psychophysiological activation in therapist-client interaction. *Front. Psychol.* 9:530. 10.3389/fpsyg.2018.00530 29695992 PMC5904261

[B110] Wegner D. M. Schneider D. J. Carter S. R. White T. L. (1987). Paradoxical effects of thought suppression. *J. Personal. Soc. Psychol.* 53 5–13. 10.1037/0022-3514.53.1.5 3612492

[B111] Weiste E. Peräkylä A. (2014). Prosody and empathic communication in psychotherapy interaction. *Psychother. Res.* 24 687–701. 10.1080/10503307.2013.879619 24517281

[B112] Westbrook D. Kennerley H. Kirk J. (2011). *An Introduction to Cognitive Behaviour Therapy: Skills and Applications.* London: Sage Publications.

[B113] Wynn R. Wynn M. (2006). Empathy as an interactionally achieved phenomenon in psychotherapy. Characteristics of some conversational resources. *J. Pragmat.* 38 1385–1397.

[B114] Young J. E. Klosko J. S. Weishaar M. E. (2003). *Schema Therapy: A Practitioner’s Guide.* New York, NY: Guilford Press.

